# PP24 Creating A Comprehensive Open-Access Health Technology Assessment (HTA) Policy Research Database Through Automated Data Extraction From HTA Reports

**DOI:** 10.1017/S0266462324001971

**Published:** 2025-01-07

**Authors:** Jan-Willem Versteeg, Lourens Bloem, Christine Leopold, Marieke De Bruin, Aukje Mantel-Teeuwisse, Wim Goettsch

## Abstract

**Introduction:**

Findable, structured, and understandable data from health technology assessment (HTA) reports is the core of HTA policy research. Available databases with this information, such as the International Network of Agencies for Health Technology Assessment (INAHTA) database, may be incomplete and their common manual data collection is time-consuming. Automated data extraction may offer a solution by creating a standardized, real-time-updating, comprehensive, open-access HTA database.

**Methods:**

In this research, we explore the possibilities of automated data extraction in the context of creating a standardized and comprehensive HTA policy research database. Data points were extracted from publicly available guidance reports of the National Institute for Health and Care Excellence (NICE) using different text extraction techniques such as natural language processing (NLP) and generative pre-trained transformers (GPTs). Future efforts are aiming to expand the database to other HTA bodies and link it to the European Medicines Regulatory Database (EMRD) that is also being developed.

**Results:**

Preliminary results of our research show that it is possible to use existing text extraction techniques to extract relevant information from publicly available HTA recommendations. Scaling the system to include more HTA bodies and data points is challenging as extraction based on document structure is complicated by heterogeneity in document structure within HTA bodies and between HTA bodies. Future results will focus on finding the best data extraction approach for each data point and on validating the system.

**Conclusions:**

Using automated data extraction to extract data from HTA reports can be a viable option for creating a comprehensive database that can be used to enhance comparative HTA policy research. Challenges remain in scaling the system to include more HTA bodies and data points. Results regarding best-performing extraction techniques and data validation of the system are expected soon.

